# Fitness peaks of dengue virus populations

**DOI:** 10.1371/journal.pone.0189554

**Published:** 2018-01-02

**Authors:** Wen Jun Liu, John G. Aaskov

**Affiliations:** 1 Australian Army Malaria Institute, Weary Dunlop Drive, Enoggera, Brisbane, Australia; 2 Institute of Health and Biomedical Innovation, Queensland University of Technology, Kelvin Grove, Brisbane, Australia; University of Hong Kong, HONG KONG

## Abstract

The role of intra-host genetic diversity in dengue viral populations remains a topic of debate, particularly the impact on transmission of changes in this diversity. Several approaches have been taken to increasing and decreasing the genetic diversity of populations of RNA viruses and have drawn what appear to be contradictory conclusions. A 2–6 fold increase in genetic diversity of a wild type population of dengue virus serotype 1(DENV1) and of an infectious clone population derived from the wild type population, produced by treatment with nucleotide analogue 5 fluorouracil (5FU), drove the populations to extinction. Removal of 5FU immediately prior to extinction, resulted in a return to pre-treatment levels of fitness and genetic diversity, albeit with novel single nucleotide polymorphisms. These observations support the concept that DENV populations exist on fitness peaks determined by their transmission requirements and either an increase or a decrease in genetic diversity may result in a loss of fitness.

## Introduction

Dengue is a disease of humans caused by at least four serotypes (1–4) of a mosquito-borne virus of the same name (dengue virus [DENV]). Not all those infected with these viruses develop clinical symptoms and only a small proportion of the 300–400 million estimated annual human infections with dengue viruses [1] result in disease severe enough to immobilise a patient. There is an extensive range of inate and acquired human host factors that may play a role in determining whether or not an infection with DENV results in clinical disease [2–5] and there are factors associated with survival of the virus that favour mild or unapparent infections in the human hosts [6,7].

The absolute measure of the fitness of a viral population is its ability to infect a new host. Changes in a virus/viral population that favour transmission in one setting may restrict transmission in another. Conditions which favour transmission are referred to as fitness peaks and those that constrain it are referred to as troughs. Collectively this is referred to as a fitness landscape[8,9]. The difficulty in measuring transmission of a pathogen between human hosts in a controlled setting has resulted in titres of viruses/bacteria/parasites in single *in vitro* systems being employed as a surrogate measure of fitness.

Not only is it in the best interest of a pathogen not to kill its host, unless transmission occurs before death, but mild disease allows the host to remain mobile and to aid dissemination of the pathogen. It has been argued that the extensive genetic diversity observed in DENV populations, much of which may be deleterious [10,11] plays an important role in dampening disease severity and allowing mobility of viraemic patients in order for virus to be disseminated more effectively than by *Aedes* mosquitoes which may travel only 50–100 metres in their lifetime [7, 12, 13]. These observations, that extremely diverse DENV populations are associated with mostly mild disease, appear to be at variance with those which demonstrated that increasing the fidelity of viral RNA-dependent RNA polymerases resulted in populations of viruses with *reduced* genetic diversity and also an attenuated phenotype [14–16]. In order to explore these issues, we examined the effects of increasing the diversity of an isolate of DENV1 from a dengue patient and of a DENV1 population derived from an infectious clone corresponding to the genome of a single virion in this natural population.

## Materials and methods

### Cells and viruses

C6-36 (mosquito) and BHK-21 (hamster kidney) cells were purchased from the American Type Culture Collection (ATCC) and cultured in 10% v/v heat inactivated foetal calf serum (FCS, Life Technology, USA)/RPMI 1640 medium (Sigma, USA). DENV1 (Myanmar 49440) was isolated from a patient by culturing 100μl of serum on C6-36 cells at 30°C for 7 days. The virus was amplified by one further passage in C6-36 cells and culture supernatant (termed WT virus) stored at -80°C.

### Construction of DENV1 plasmids and generation of infectious virus

The DENV1 infectious clone was produced using a two plasmid ligation strategy [17]. RNA was extracted from WT DENV1 (QIAamp Viral RNA Mini kit, Qiagen, USA) and reverse transcribed using random hexanucleotide primers (Promega, USA) and Superscript II Reverse Transcriptase (Invitrogen, USA) according to the manufacturer’s instructions. cDNA was amplified using high fidelity *Pfu* DNA polymerase (Promega, USA) and cloned into a single-copy bacterial artificial chromosome plasmid pBeloBac11 (New England Biolabs, USA) and sequenced. The DENV1 cDNA fragments in cloned plasmids were head-tail ligated to construct two plasmids. The first plasmid contained cDNA corresponding to the region of the DENV genome from the 5’UTR terminal to an *AgeI* restriction enzyme site at nucleotide 5813. A *SmaI* restriction enzyme site and a T3 promoter sequence were introduced upstream of the DENV1 5’UTR and a *SacII* restriction enzyme site was added following the DENV1 *AgeI* restriction enzyme site. A second plasmid contained cDNA corresponding to the region of the DENV genome from nucleotides 5813 (*AgeI*) to the 3’ end of the 3’UTR followed by a *SacII* restriction enzyme site.

Both plasmids were digested with *AgeI* and *SacII* restriction enzymes and ligated to form full length DENV1 cDNA. The infectious clone virus was rescued by electroporation of *in vitro*-transcribed RNA into BHK-21 cells as published methods [18]. Virus in the supernatant from cultures of transfected cells was passaged a second time, in C6-36 cells, to produce virus of sufficient titre for subsequent experiments.

### Infection of cells, 5-fluorouracil (5FU) treatment and plaque assays

5FU (Sigma, USA) was dissolved in dimethylsulphoxide according to the manufacturer’s instructions, sterilized by filtration through a 0.2μm filter and stored at -20°C. Confluent monolayers of C6-36 cells in 12 well plates (Falcon, USA) or T-25 flasks (Falcon, USA) were incubated with 5% v/v FCS-RPMI 1640 medium containing 5FU, at the concentrations indicated in the text, for 3 hours at 30° C. Cell culture fluid was removed and cells were infected with DENV1 at the multiplicities of infection (MOI) indicated in figure legends. Cell culture fluid was removed 3 hours post-infection, the monolayer washed with 2% v/v FCS-RPMI 1640 and fresh 5% v/v FCS-RPMI 1640 medium, with or without 5FU, added. Cultures were incubated at 30° C in an atmosphere of 5% v/v CO_2_/air and culture fluid was harvested at time points thereafter. The titre of infectious virus in both 5FU-treated and untreated samples was determined using a standard DENV immuno-plaque assays (indirect ELISA). Briefly, monolayers of C6-36 cells in 12 well plates were infected with 10-fold dilutions of virus (100μl) and incubated at 30°C in an atmosphere of 5% v/v CO _2_ / air. After 2 hours, the culture fluid was removed and the cell monolayers overlaid with 2 ml 5% v/v FCS, 1.5% v/v carboxy-methyl cellulose (CMC; Sigma, USA) /RPMI medium. Six days after infection, cell monolayers were fixed with 5% v/v formaldehyde-PBS for 10 minutes, and permeabilised with 0.5% v/v Triton X-100 (Sigma, USA)/PBS and stained with horseradish peroxidase-conjugated anti-DENV monoclonal antibodies [19]. After washing the cell monolayers with 0.5% v/v Tween 20 (Merck, USA)/PBS, plaques were visualised by adding 3,3,5,5 tetramethylbenzidine (TMB; Sigma, USA) and counted. Mock-infected cultures, maintained in parallel, were used as controls.

### Cloning and sequencing

Oligonucleotide primers D1-843F (5’- ATGCCATAGGAACATCC-3’) corresponding to genome position 827–843 of the DENV1 genome and D1-2465R(5’- ACTTCATTGGTG ACAAAAATGCC-3’) corresponding to genome position 2471–2493 were used to amplify the DENV1 envelope (E) gene from cDNA. PCR was performed with 5μl 10× high fidelity polymerase buffer (Roche, Germany), 250ng of both forward and reverse primers, 1μl 10 mM deoxynucleoside triphosphates (dNTPs) (Promega, USA), 2.5 U Expand High Fidelity PCR polymerase (Roche, Germany), 5μl of cDNA and RNase-free water to a final volume of 50μl. PCR thermocycler conditions were 94°C for 1 min, followed by 36 cycles of 94°C for 30s, 54°C for 30s, and 70°C for 1.5 min, and 70°C for 10 min. PCR products were visualized following electrophoresis on a 1% (w/v) agarose TAE gel using ethidium bromide and UV light.

PCR amplicons were purified using MinElute Gel Extraction kits (Qiagen, Germany) and cloned into the T/A cloning vector pGEM-T Easy (Promega, USA) according to the manufacturer’s instructions. E gene consensus sequences were derived from RT-PCR cDNA fragments using the PCR primers D1-843F and D1-2465R (above).

Sequencing was performed at the Australian Genome Research Facility by dye di-deoxy chain termination. E gene inserts in 19–25 clones were sequenced to estimate the genetic diversity in each sample. The mutation frequencies were calculated as total number of mutations/total number of nucleotides sequenced. Sequences have been submitted to Genbank (Infectious clone virus: JF459993, Wild Type: AY726553).

Neighbour Joining phylogenetic analyses, employing the Jukes Cantor genetic distance model, were performed with the sequences of E genes derived from cDNA cloned into pGEM-T easy plasmids, above. Similar trees were obtained using PHYML and the GTR substitution model. Analyses were performed with Geneious software purchased from Biomatters Ltd.

### DENV 1 RNA quantification

DENV1 viral RNA copy numbers were determined using real-time PCR on a Mx4000 multiplex PCR system (Stratagene, USA), a SYBR green-based assay and DENV1 primers DENV1F (5’-GGRACKCAGGWTCTCC-3’) corresponding to genome position 4898–4915 and DENV1R (5’AGTTTCTTTTCCTAAACACCTCG-3’) corresponding to genome position 5067–5045. A plasmid containing an insert corresponding to this region was used as a standard. Negative controls (without template cDNA) were included with each amplification reaction. Denaturation curves were used to monitor the specificity of the amplification. Each value for the amount of DENV1 RNA is the average of at least three determinations. Results were expressed as cDNA copy number per μl of cDNA.

### Viral RNA infectivity

The infectivity of viral RNA was determined by electroporating 5x10^8^ molecules of DENV RNA into C6-36 cells in 0.4 cm cuvettes (Bio-Rad, USA) using square wave pulses of 130V for 25ms in a Bio-Rad gene pulser Xcell system. Electroporated cells were incubated in 25 cm^2^ flasks in 10% v/v FCS–RPMI 1640 medium at 30°C in 5% v/v CO_2_/air for 4 hours before the medium was aspirated and replaced with 10% v/v FCS-RPMI 1640 medium. At the times shown in the text, cell culture supernatant was harvested and infectious DENV quantified by plaque assay on C6-36 cells as described above.

## Results

**Fig 1:**
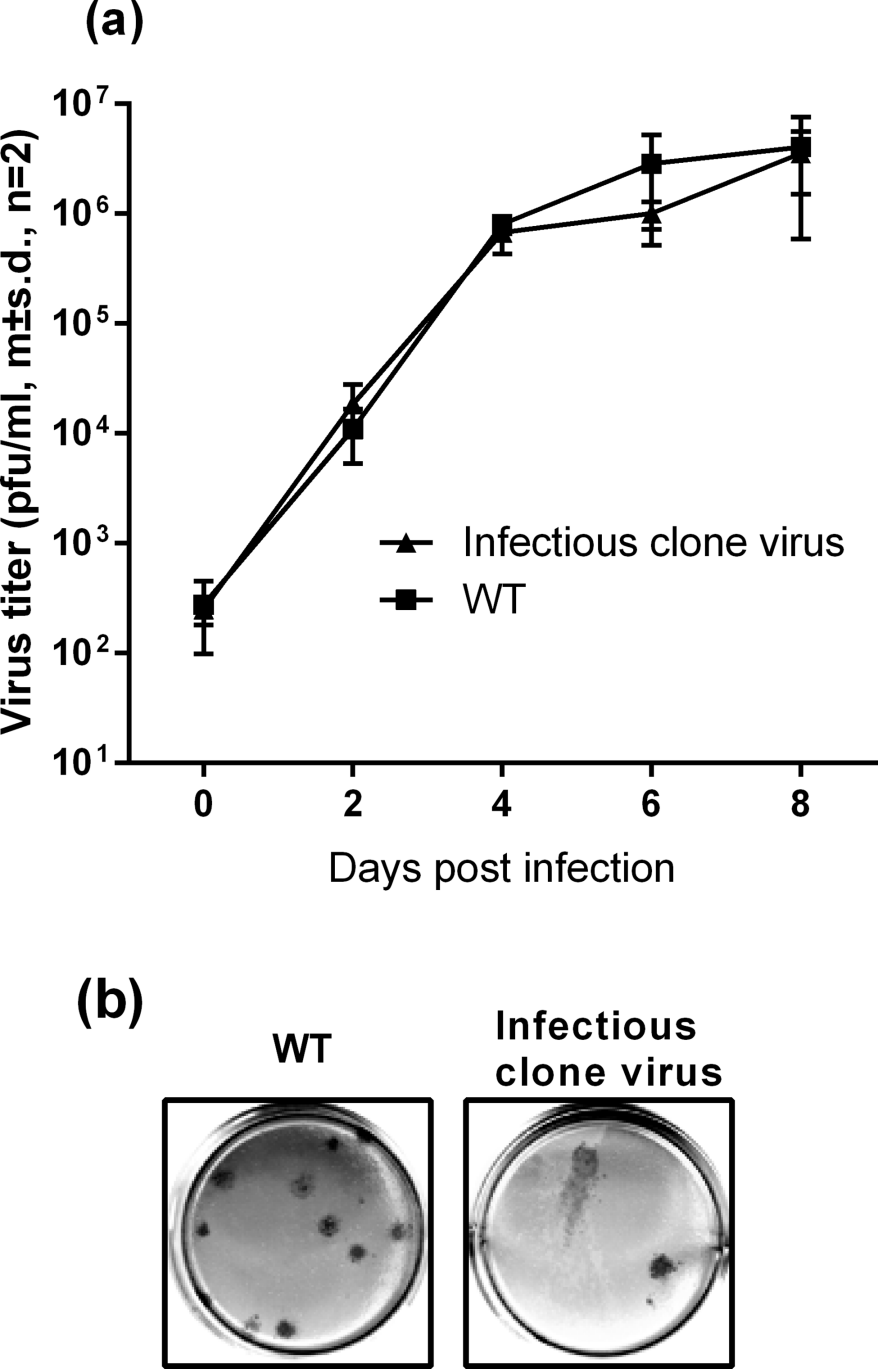
Growth kinetics and plaque morphology of wild type (WT) and infectious clone-derived DENV1 in C6-36 mosquito cells. (a) Yield of virus after C6-36 cells were infected at an MOI of 0.1 and (b) plaque morphology identified by indirect ELISA seven days post-infection.

**Fig 2:**
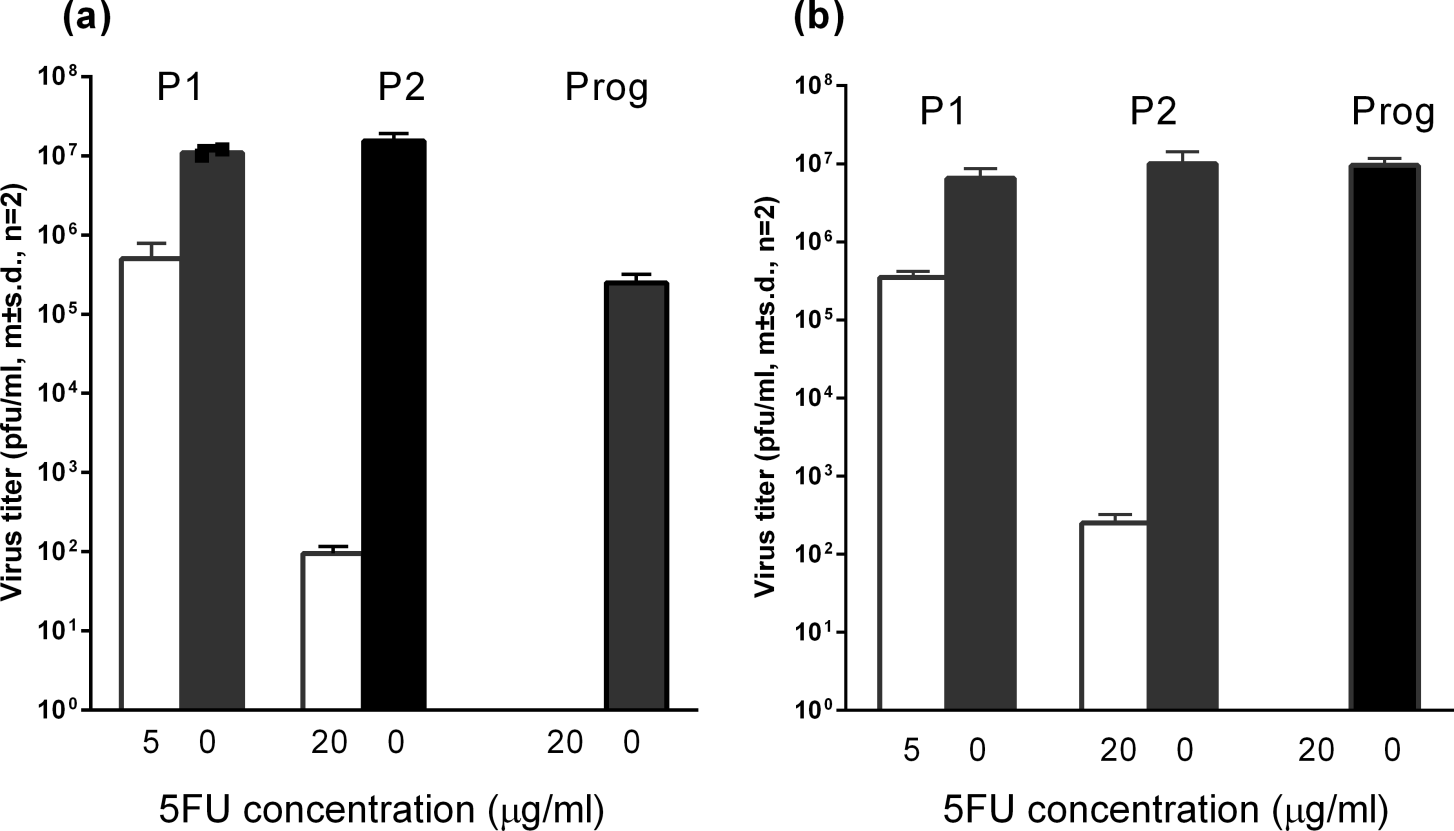
Titres of extracellular virus in cultures of C6-36 cells 7 days after infection with (a) WT DENV1 or (b) DENV1 derived from an infectious clone of the WT virus. Virus (culture medium) from cultures treated with 5μg/ml 5FU (P1, open bars) was transferred to cultures of C6-36 cells in the presence (solid bars) or absence (open bars) of 20 μg/ml 5FU. Virus (culture medium) from cultures treated with 20 μg/ml 5FU (P2, open bars) was transferred to cultures of C6-36 cells in the presence (open bars) or absence (solid bars) of 20 μg/ml 5FU. Prog.–progeny of the pre-extinction stocks of virus.

No significant differences were observed between the growth kinetics (Fig 1A) or plaque morphology (Fig 1B) in C6-36 cells of a DENV1 isolate (WT) and virus generated from an infectious clone of this isolate. Infection of cultures of C6-36 cells with the WT and infectious clone-derived DENV1, at an MOI of 0.1, in the presence of 5FU resulted in similar reductions in virus yield (P1, Fig 2). The 5FU treated DENV1 (culture supernatant from P1, open bar) then was used to infect new cultures of C6-36 cells in the presence of 20μg/ml 5FU (Fig 2, P2, open bars) or absence of 5FU (Fig 2, P2 black bars). In the absence of 5FU, yields of DENV1 returned to pre-treatment levels while the presence of 5FU resulted in a further reduction in titres of infectious virus (P2, Fig 2A and 2B). Passage of the P2 5FU-treated virus in C6-36 cells the absence of 5FU resulted in titres the same as, or similar to, those observed with untreated virus (Prog., “progenitor”, closed bars, Fig 2A and 2B) while no virus production could be detected in cultures of cells infected with these virus stocks in the presence of 20μg/ml 5FU. Virus recovered after the second passage in the presence of 5FU was labelled “pre-extinction virus”.

**Fig 3:**
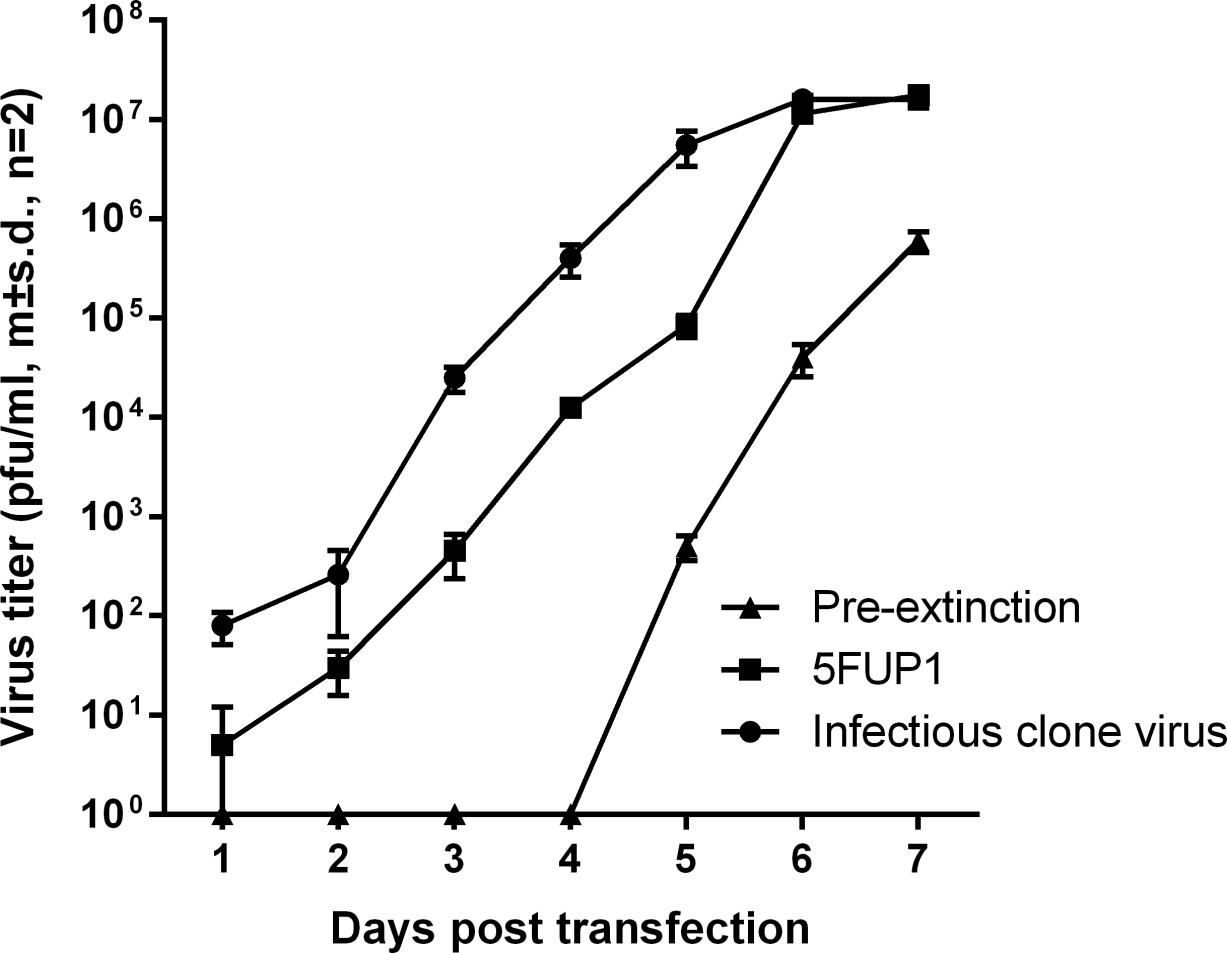
Kinetics of DENV production by cultures of C6-36 cells transfected with 5 x 10^8^ molecules RNA extracted from initial stocks of infectious clone derived DENV1 (IC), from IC passaged once in the presence of 5 μg/ml 5FU (5FUP1; P1, Fig 2B) and from IC passaged twice in the presence of 5FU (Pre-extinction; P2, Fig 2B).

In order to distinguish between any non-specific toxicity of 5FU for cells infected with DENV1 and the direct effect on the genomes of DENV, RNA was extracted from untreated infectious clone derived virus, from virus produced after one cycle of 5FU-treatment (P1, Fig 2B) and from pre-extinction virus (P2, Fig 2B) and used to electroporate C6-36 cells in the absence of any 5FU. While the yield of infectious DENV1in the supernatant of cultures electroporated with RNA from DENV1 produced in the presence of 5ug/ml was the same as that from cultures electroporated with RNA from untreated DENV1 after 6 days (Fig 3) the kinetics of production were slower. In the case of RNA from DENV1 recovered after two cycles of 5FU treatment, the kinetics of production of infectious DENV1 from electroporated cells was slower and the final yield lower than from cultures of cells electroporated with RNA from untreated virus or from virus cultured only once in the presence of 5FU. These results suggested that 5FU was having a direct, and dose-dependent, effect on the RNA being incorporated into DENV1 virions.

**Fig 4:**
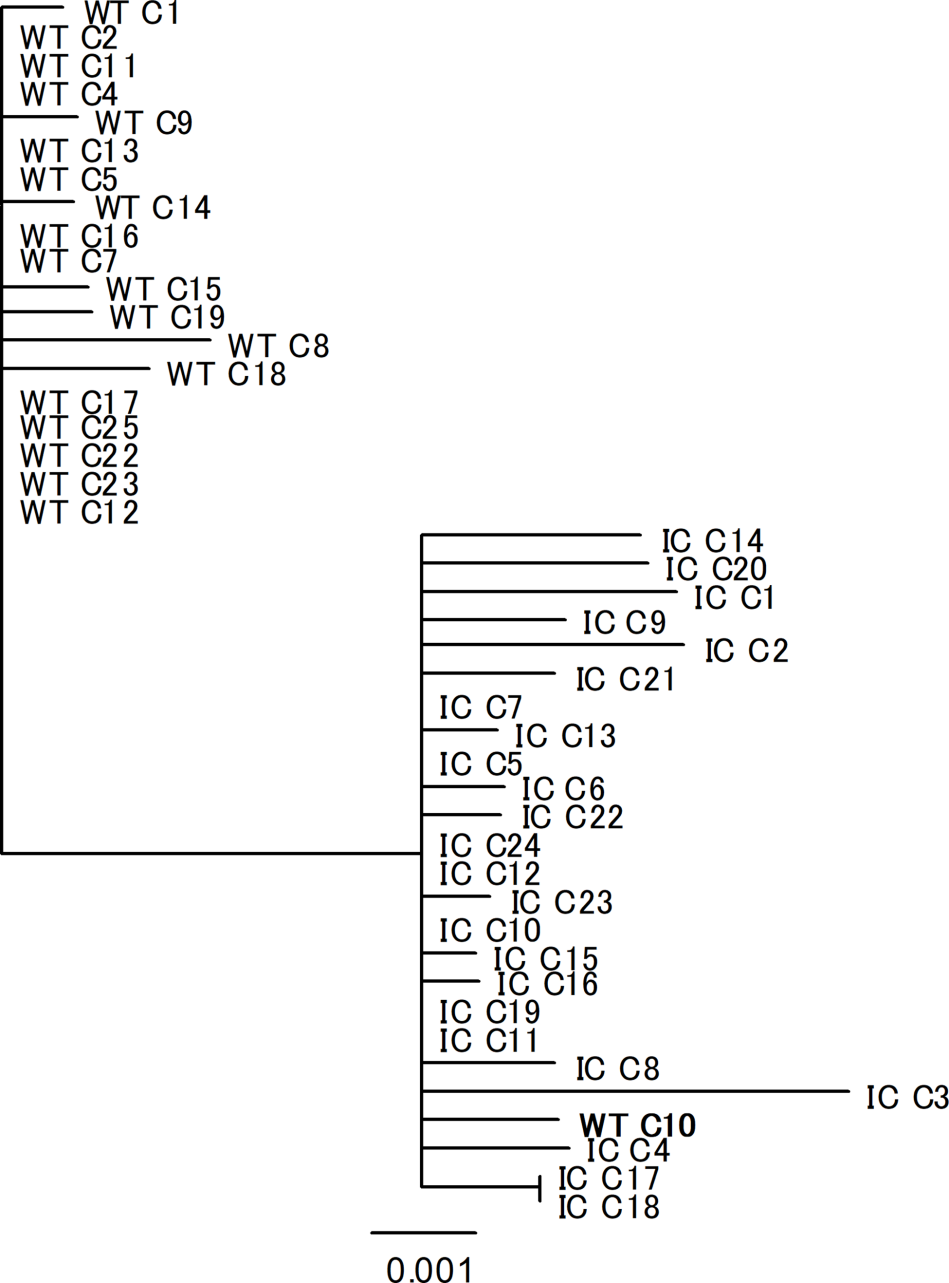
Phylogenetic relationships between the envelope (E) protein genes of members of the initial stocks of wild type (WT) and infectious clone (IC) derived DENV 1. The bar represents the number of nucleotide substitutions per site.

E genes of the WT and infectious clone-derived DENV1 populations were amplified by RT-PCR and the resultant cDNA cloned and sequenced. The consensus sequence for each of the three WT and each of the three infectious clone derived DENV1 populations, shown in Table 1, were the same. While the WT population appeared to be more homogeneous than the infectious clone derived one (i.e. proportion of clones sharing the consensus sequence; mutation frequency), the differences were not significant (p> 0.05, Chi ^2^ test). The contribution of PCR errors to this and subsequent measurements of diversity was estimated by PCR amplification of the DENV1 E gene from a plasmid, ligating the cDNA into a new plasmid, transforming *E*. *coli* and then sequencing the plasmids recovered from eighteen bacterial colonies. The contribution of errors from this source to subsequent estimates of genetic diversity would have been minimal (Table 1). The separation of the WT clade and the infectious clone derived clade on a phylogenetic tree (Fig 4.) and the presence of a single WT sequence (**WT C10**) in the infectious clone clade, suggested that the infectious clone had been derived from a minority member of the WT population.

**Table 1:** Genetic diversities of input, 5FU-treated and their progeny DENV 1 populations.

**Strain**	**No. clones** **sequenced**	**No. clones** **with** **consensus** **sequence**	**No.variable** **nt. sites**	**No. variable a.a. sites**	**No. n.t. changes**	**No. a.a. changes**	**Mutation frequency**
PCR control	18	15	2	2	2	2	7.50 x 10^−5^
Infectious clone virus	24	7	35	24	35	24	9.82 x 10^−4^
Infectious clone virus pre- extinction	24	2	61	41	71	50	1.99 x 10^−3^
Infectious clone virus progeny	20	1	28	17	39	28	1.31 x 10^−3^
WT virus	22	12	19	12	19	12	5.82 x 10^−4^
WT virus pre- extinction	23	0	65	40	127	65	3.72 x 10^−3^
WT virus progeny	24	8	24	13	32	17	8.98 x 10^−4^

**Fig 5:**
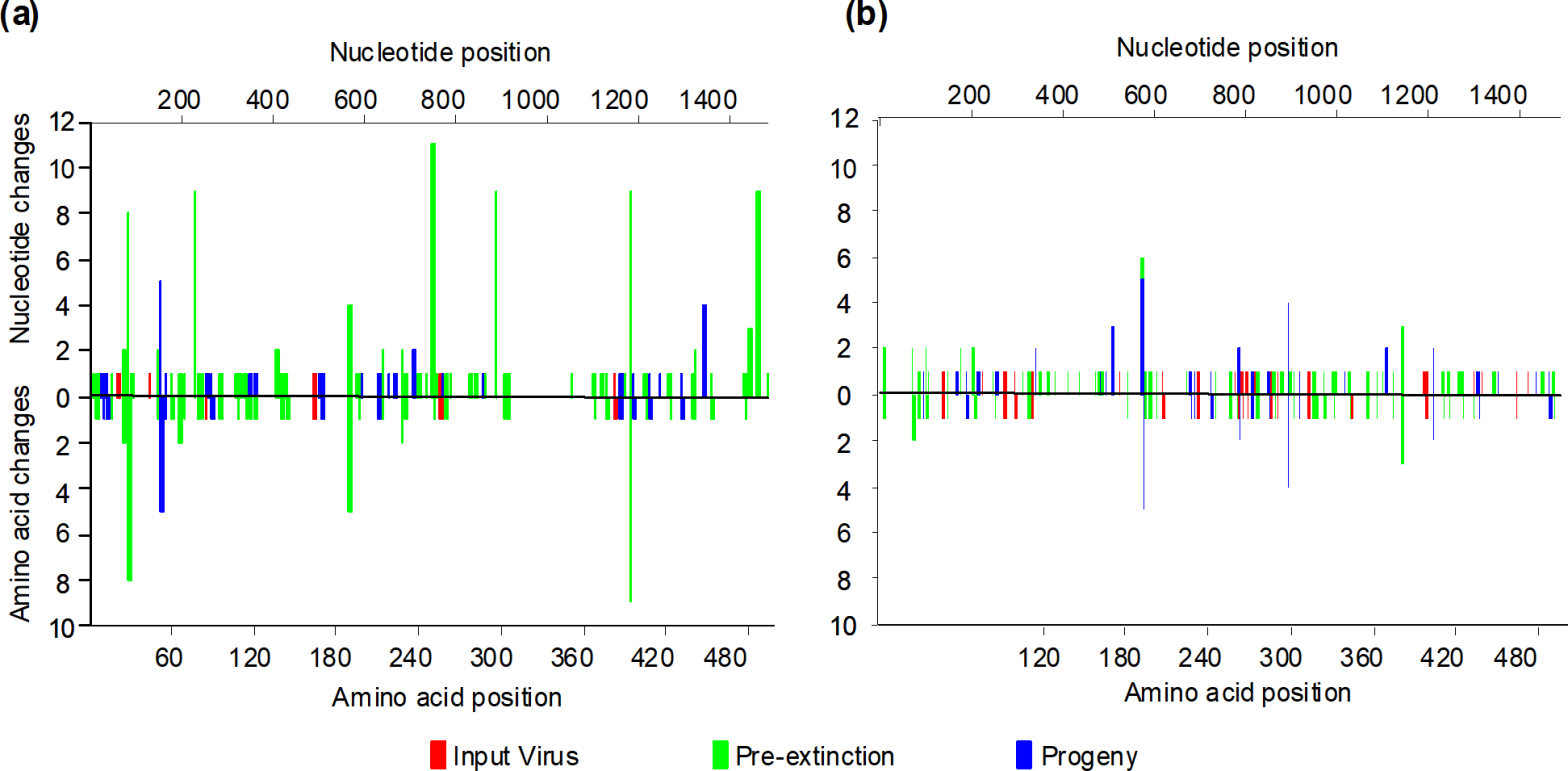
Distribution of variable sites across the envelope protein genes of (a) wild type and (b) infectious clone derived DENV1 populations prior to 5FU treatment (red), after two passages in 5FU (green; P2, pre-extinction populations, Fig 2) and after recovery from 5FU treatment (blue; progeny [Prog] population, Fig 2).

Two passages of WT and infectious clone-derived DENV1 in the presence of 5FU brought the populations to the verge of extinction (Fig 2) and resulted in a significant increase (p < 0.001, Chi^2^ test) in the number of nucleotide and amino acid polymorphisms in these populations (Table 1). However, a single passage of these two pre-extinction populations (P2, Fig 2), which were unable to withstand further treatment with 5FU, in the absence of 5FU, reduced this diversity to pre-treatment levels i.e. there was no significant difference (p> 0.05, Chi^2^ test) between the diversity of untreated populations of virus and these progeny from the pre-extinction populations (Progeny, Fig 2; Table 1). While some of the return to pre-treatment levels of diversity was due to reversion to original sequence, there also were new single nucleotide polymorphisms (SNPs) in the progeny populations. The increase in genetic diversity associated with exposure to 5FU and the novel polymorphisms in progeny viral populations did not result in a change in the consensus sequence. The number and location of polymorphisms in the E gene of the two DENV1 populations accompanying the treatment with and recovery from 5FU exposure are shown in Fig 5. The increase in diversity in the 5FU treated WT population appeared to arise by a large number of changes at a few sites while that in the infectious clone derived population arose by a small number of changes at a larger number of sites.

**Fig 6:**
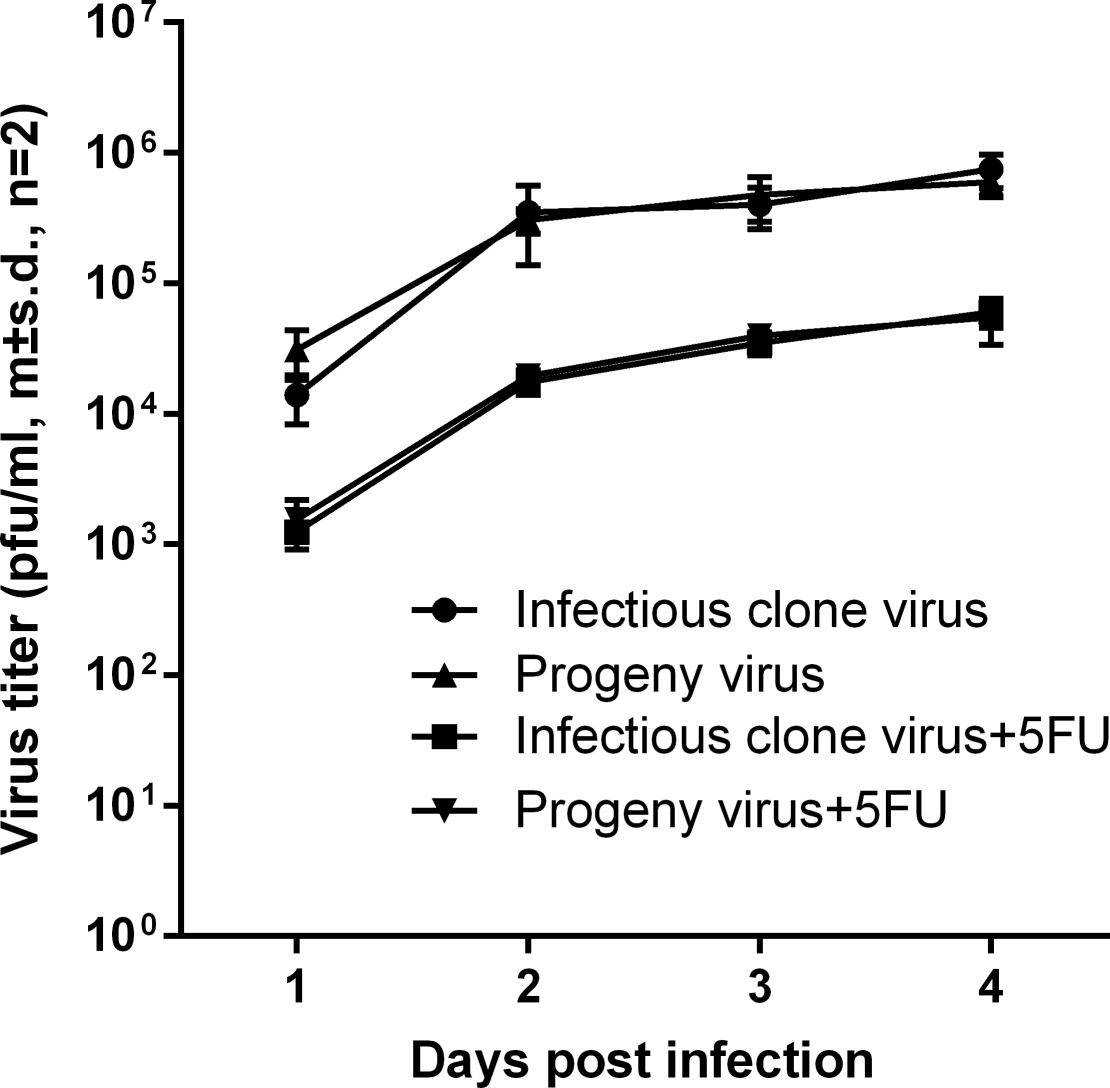
Effect of 5FU (20 μg/ml) on the yield of virus in cultures of C6-36 cells infected at an MOI of 0.1 with infectious clone derived DENV1 or the progeny of the pre-extinction population of infectious clone derived DENV1 (open bar, P2, Fig 2B).

The novel polymorphisms which arose in the infectious clone derived virus population during treatment with 5FU and subsequent recovery (“progeny” virus) did not confer resistance to subsequent exposure to 5FU (Fig 6) i.e. the reduction in yield of infectious virus in cultures treated with 20 μg/ml 5FU was similar for the original stock of infectious clone derived DENV1 and for the progeny virus derived from 5FU treated stock (P3, Fig 2B).

## Discussion

The aims of this study were to determine whether an homogenous DENV1 population (derived from an infectious clone) would gain or lose fitness as it approached the diversity of the naturally occurring population from which it was derived and to then determine the effect of increasing the diversity of both populations. For the purpose of this study, we have defined fitness as the yield of virus from cells infected in a culture and acknowledge that this ignores the critical component of transmission ability. However, transmission is, almost certainly, influenced by the level of viraemia in a patient and the titre of virus in mosquito saliva. It was not possible to address all these aims because DENV derived from cells electroporated with RNA from a single plasmid was as diverse as the natural, un-cloned, population of DENV from which it was derived (Table 1). Although unanticipated, it is perhaps not surprising that the magnitude of the diversity in the population derived from a DENV1 infectious clone was similar to that isolated from a patient given that there must be several thousand rounds of replication between transfection of cells with DENV RNA and the generation of high titre working stocks in the C6-36 mosquito cells, and each round of replication with a predicted error rate of between 10^−3^ to 10^−5^ nucleotide substitutions [20].There would have been limited opportunities for selective sweeps to remove mutations during preparation of working stocks of DENV from the infectious clone compared to those available in natural cycles of transmission. Furthermore, both populations of DENV1 demonstrated similar initial fitness in cultures of mosquito cells i.e. they produced similar sized plaques and replicated at similar rates and to similar titres (Fig 1). However, while the overall genetic diversity was similar, the structure of these two populations was different (e.g. branch lengths tended to be shorter in phylogenetic trees constructed using infectious clone derived sequences than WT sequences) perhaps reflecting the different selection pressures shaping a DENV population in cell culture and those operating in natural cycles of transmission.

Increasing the genetic diversity in these two populations of DENV1 from 2–6 fold (Table 1) resulted in a loss of fitness of more than 1000 fold as measured by the yield of virus from infected mosquito cells (Fig 2). The magnitude of the increases in diversity that could be tolerated before these populations became extinct were similar to that reported for other RNA virus populations [21]. The ability of the WT population to tolerate greater change (more than six fold, Table 1) than the infectious clone virus (approximately two fold, Table 1) and still replicate in mosquito cells *in vitro* may reflect a plasticity required to enable it to cycle between human hosts and mosquito vectors in nature.

It was not expected that removal of the mutagen (5FU) would result in such a rapid rebound to pre-treatment levels of diversity and fitness in these two populations (Table 1, Fig 2). Several observations flow from this. The first is that the diversity of naturally occurring DENV populations falls within a relatively narrow range [10,22–25] and increasing this resulted in a rapid loss of fitness. Return to pre-existing fitness levels was accompanied by a return to “natural” levels of genetic diversity but with a different range of SNPs to that found in the original populations. This suggests that the fitness of a DENV population may depend as much on the magnitude, as the nature, of the within-population genetic diversity. It is difficult to explain why the *magnitude* might be important, particularly with synonymous changes, unless they were providing flexibility in codon usage as the population moved between host and vector or variability in the secondary structure of viral RNA that enabled efficient binding of both mosquito and human host cell proteins. Significantly, none of these changes due to treatment with 5FU, or the subsequent recovery when this agent was removed, resulted in a change in the consensus nucleotide sequence of the virus populations i.e. there were significant changes in fitness without a change in consensus sequence. These observations highlight the risks associated with attempting to define a DENV phenotype based on the consensus genotype of the population.

The gain in fitness (yield of virus in culture) which accompanied the loss of diversity in the 5FU-treated, pre-extinction populations, of DENV1 and the return to pre-treatment levels of diversity would appear to contrast with the loss of fitness observed with populations of virus with high fidelity RNA dependent RNA polymerases that restricted population diversity [14–16]. However, this apparent contradiction disappears if fitness is considered as a landscape with various peaks [26,27] on which virus populations exist as a result of adaption to their natural cycles of transmission. In this model, either an *increase* or a *decrease* in population diversity could result in a loss of fitness. The mechanisms by which DENV population diversity might be maintained within the relatively narrow limits necessary for the populations to remain at, or near, their peaks of fitness remain unclear. Population bottlenecks and “selective sweeps” have been proposed but the mechanisms by which a selective sweep acts and the nature of the target remain far from clear.
